# Supplementary material for: COVID-19 Disease in Pediatric Solid Organ Transplantation from Alpha to Omicron: A High Monocyte Count in the Preceding Three Months Portends a Risk for Severe Disease
Source: Viruses. 2023 Jul 16;15(7):1559. doi: 10.3390/v15071559 (PMC10383409; doi:10.3390/v15071559)
Supplement: Supplementary file 1 [file viruses-15-01559-s001.zip › Supplemental Figure legend.pdf]

Supplemental Figure 1. Pearson's Correlations among the observed levels of NK cell number and frequency before infection, CRP, LDH, ferritin, absolute monocyte and lymphocyte count at time of infection were computed. Blue boxes indicate a positive correlation and brown boxes indicate a negative correlation. All correlations were significant with $p<0.01$.
