# Supplementary figures and images for: COVID-19 Disease in Pediatric Solid Organ Transplantation from Alpha to Omicron: A High Monocyte Count in the Preceding Three Months Portends a Risk for Severe Disease
Source: Viruses. 2023 Jul 16;15(7):1559. doi: 10.3390/v15071559 (PMC10383409; doi:10.3390/v15071559)

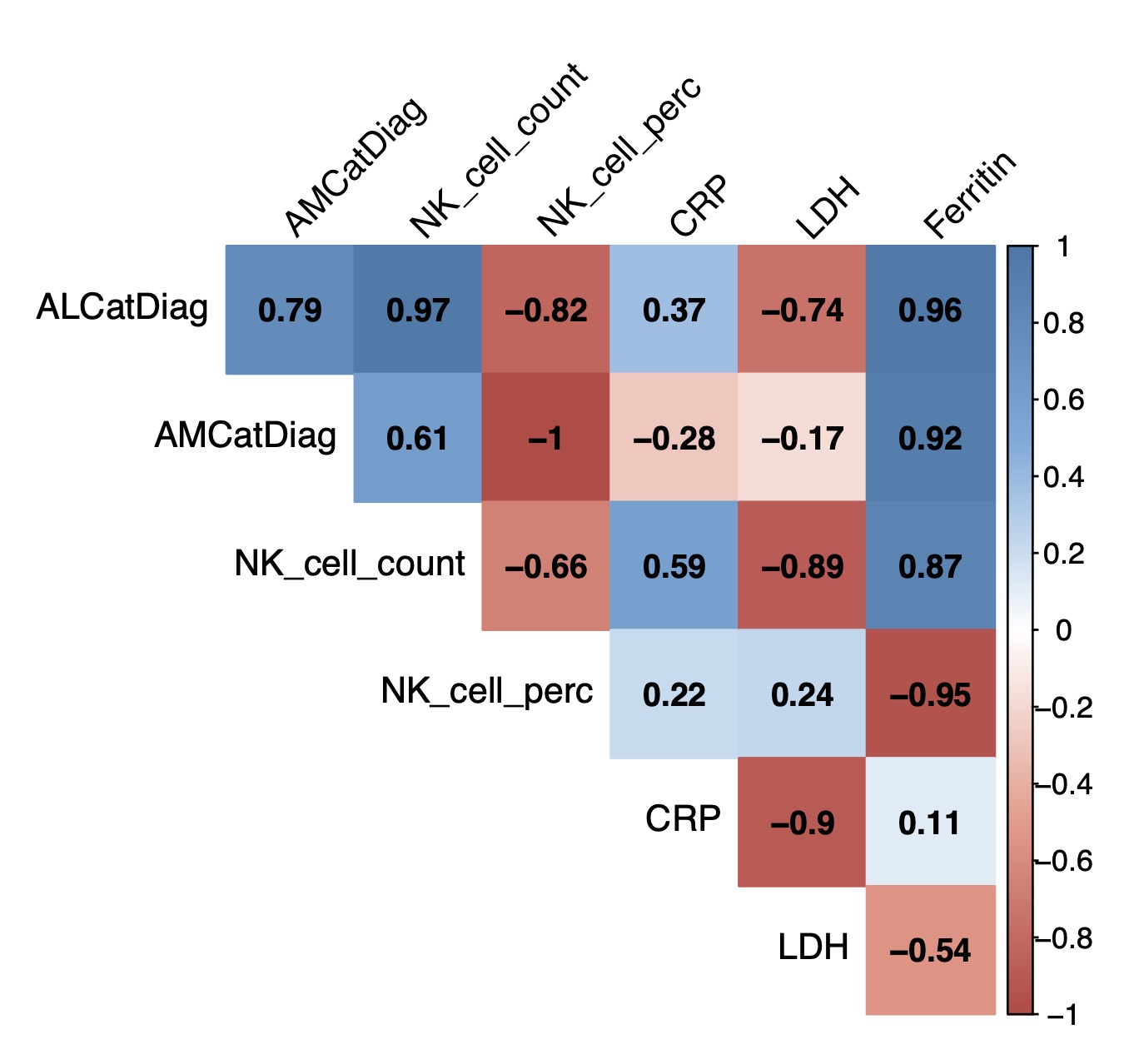

Supplement: Supplementary file 1 [file viruses-15-01559-s001.zip › Supplemental_fig 1.jpg]
